# Deciphering the role of alternative splicing as a potential regulator in fat-tail development of sheep: a comprehensive RNA-seq based study

**DOI:** 10.1038/s41598-024-52855-1

**Published:** 2024-01-29

**Authors:** Mohammad Reza Bakhtiarizadeh

**Affiliations:** Department of Animal and Poultry Science, College of Aburaihan, University of Tehran, Tehran, Iran

**Keywords:** Bioinformatics, Gene expression analysis

## Abstract

Although research on alternative splicing (AS) has been widely conducted in mammals, no study has investigated the splicing profiles of genes involved in fat-tail formation in sheep. Here, for the first time, a comprehensive study was designed to investigate the profile of AS events and their involvement in fat-tail development of sheep. In total, 45 RNA-Seq samples related to seven different studies, which have compared the fat-tailed vs thin-tailed sheep breeds, were analyzed. Two independent tools, rMATS and Whippet, along with a set of stringent filters were applied to identify differential AS (DAS) events between the breeds per each study. Only DAS events that were detected by both tools as well as in at least three datasets with the same ΔPSI trend (percent spliced in), were considered as the final high-confidence set of DAS genes. Final results revealed 130 DAS skipped exon events (69 negative and 61 positive ΔPSI) belonged to 124 genes. Functional enrichment analysis highlighted the importance of the genes in the underlying molecular mechanisms of fat metabolism. Moreover, protein–protein interaction network analysis revealed that DAS genes are significantly connected. Of DAS genes, five transcription factors were found that were enriched in the biological process associated with lipid metabolism like “Fat Cell Differentiation”. Further investigations of the findings along with a comprehensive literature review provided a reliable list of candidate genes that may potentially contribute to fat-tail formation including HSD11B1, SIRT2, STRN3 and TCF7L2. Based on the results, it can be stated that the AS patterns may have evolved, during the evolution of sheep breeds, as another layer of regulation to contribute to biological complexity by reprogramming the gene regulatory networks. This study provided the theoretical basis of the molecular mechanisms behind the sheep fat-tail development in terms of AS.

## Introduction

Sheep breeds can be divided into three major groups in terms of tail morphology, fat-tailed, thin-tailed and fat-rumped^1^. Fat-tailed breeds constitute ~ 25% of the world’s sheep breeds^1,2^. It is believed that fat-tailed breeds have evolved through natural and artificial selection, after initial domestication of their wild ancestors, for adaptation to harsh and difficult environmental conditions (such as drought and periodic food shortages)^3^. However, fat-tail has lost its advantages in the current commercial production systems, as it influences mating, locomotion and carcass characteristics; requires more energy to produce and currently consumers prefer low-fat food^3–6^.

Investigation of genetic factors affecting complex traits, such as fat metabolism, based on monitoring the underlying molecular mechanisms is of major interest among scientists. Understanding these mechanisms can dramatically accelerate genetic improvement. In this regard, numerous efforts have been made to better understand the genetic background of fat deposition in tail of sheep, such as^6–10^, which can be applied to evolve new and improved sheep breeds. A literature review showed that most of these studies focused on comparative transcriptome/variant analysis between fat- and thin-tailed sheep breeds^5–7,9–16^. In this context, our team previously conducted a comprehensive gene expression meta-analysis of six independent RNA-Seq datasets that compared transcriptome profiles of fat- and thin-tailed sheep breeds and a core set of differentially expressed genes (DEGs) as well as several pathways associated with lipid metabolism were reported^17^.

Based on the Ensemble database (release 106), transcript per gene ratio in sheep genome is ~ 1.7 (20,137 known protein coding genes and 34,569 known transcript), indicating an average of approximately two transcripts for each gene caused by alternative splicing (AS). AS is one of the fundamental regulatory features of eukaryotic cells that enables a gene to encode multiple transcripts and can lead to the enhanced diversity of transcriptome and proteome^18^. It is reported that AS can be more important than gene expression profiling in facilitating rapid adaptive divergence within short time periods^19^. Increasing evidence has highlighted the importance and the potential roles of AS in various tissue types, developmental stages and diseases^20^. For example, it has been reported that 92–94% of the human genes are alternatively spliced, as most of them are tissue-specific^21^. Moreover, differential AS events have been identified between different breeds such as cattle^22^ and chicken^23^. For instance, the identified differential AS (DAS) genes in muscle, brain and skin tissues of mules were significantly enriched in "muscle contraction" pathway^24^. Miao et al., reported 356 DAS events in comparison between muscle tissue of small-tailed Han and Dorset sheep breeds, which were associated with variations in muscle development^25^. Also, 411 DAS events were found in adipose tissue between small-tailed Han and Dorset sheep breeds^26^. These reports reinforced the fact that AS events have diverged rapidly during vertebrate evolution^27^. In this context, AS has been reported as an important source of species-specific divergence^28^. These reports have resulted in a growing interest in understanding of AS events in various biological process as well as among different breeds. Although several studies have investigated the transcriptome differences between fat- and thin-tailed sheep breeds^6,7,9–11,16^, comprehensive alterations of AS and their biological implications in fat deposition in tail of sheep has not been elucidated, yet. Fortunately, a large number of public RNA-Seq datasets provide an opportunity to investigate role of AS in gene regulation in fat-tail formation of sheep, which can be overlooked in standard transcriptome analysis. To fill this knowledge gap, a genome-wide analysis was performed to identify DAS events between fat-tail tissue of fat- and thin-tailed sheep breeds based on seven independent RNA-Seq studies. Furthermore, functional enrichment analysis was performed for a better understanding of the biological functions of the genes with identified DAS. This study tried to provide insights into the molecular basis that may contribute to fat-tail formation in sheep breeds.

## Results

### RNA-seq data analysis

In total, over 1.133 billion reads were analyzed, which were belonged to 22 fat- (totaling 556 million reads) and 23 thin-tailed (totaling 577 million reads) samples. On average, each sample contained more than 25 million reads, ranging from 7.5 to 50 million reads. After trimming, approximately two million reads were removed, indicating the high quality of the datasets. Out of a total of 1.131 billion clean reads, 1.036 billion reads (~ 92.6%) were successfully aligned to the genome. Of the aligned reads, 87.5% were uniquely mapped, while the remaining 12.5% were mapped to multiple locations in the genome. Alignment rates for individual samples ranged from 77.1 to 98.5%. The Supplementary File S1 contains a summary of the RNA-Seq datasets used in this study along with mapping results.

### Identification of DAS events by rMATS

**Figure 1:**
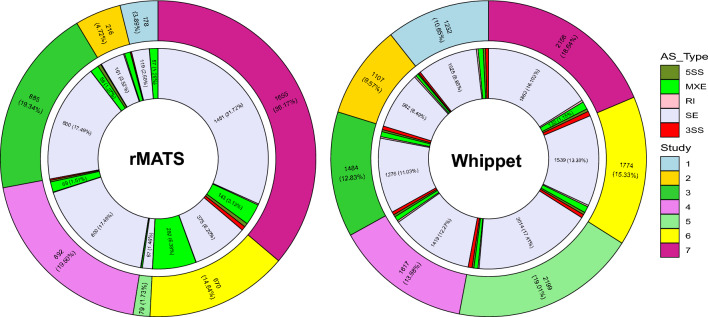
Number and percentage of the identified AS events by rMATS and Whippet tools in different studies. AS_Type indicates the different AS events including: skipped exon (SE), intron retention (IR), alternative 5′ splice site (5SS), alternative 3′ splice site (3SS) and mutually exclusive exon (MXE). Inner and outer circles indicate AS events and studies, respectively. Study 1 (PRJNA508203), Study 2 (PRJNA699984), Study 3 (PRJNA432669), Study 4 (PRJNA745517), Study 5 (PRJNA517348), Study 6 (PRJNA598581) and Study 7 (PRJNA792697).

**Figure 2:**
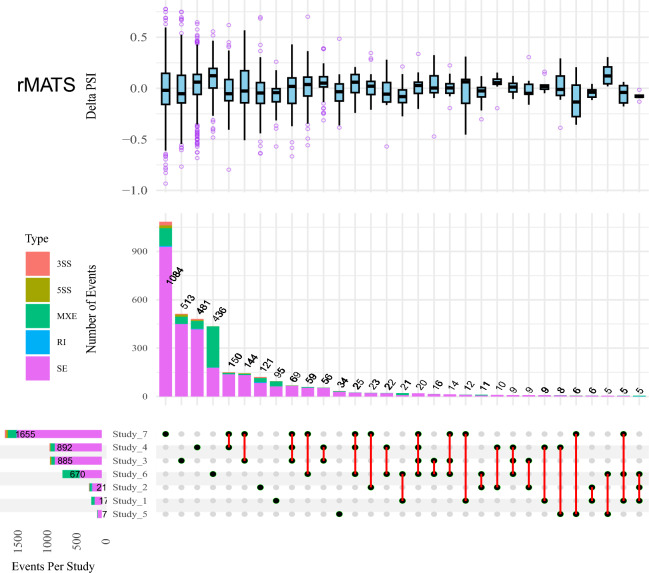
UpSet plot of the intersection of DAS events among the different studies identified by rMATS. The box plot in the top section indicates the distribution of ΔPSI in each group. The bar plot in the middle section indicates the number of event types shared by specific studies. The horizontal bar chart at the bottom left represents the total number of events found in each study. An observation threshold of five is used to exclude low-count subsets.

DAS events were identified by comparing the fat- and thin-tailed samples in each dataset. Using rMATS, 4,575 DAS were detected including 3773 skipping exon (SE), 667 mutually exclusive exon (MXE), 62 alternative 5′ splice site (5SS), 51 alternative 3′ splice site (3SS) and 22 intron retention (IR) events. The largest number of DAS events (1655) were identified in Study 7, followed by Study 4, Study 3, Study 6, Study 2, Study 1 and Study 5. The complete list of the identified DAS events by both tools is provided in Supplementary File S2. Figure 1 represents the number and percentage of different DAS events in the seven studies by the tools. The highest number of common DAS events was identified by rMATS between study 4 and 7 (150); and study 7 and 3 (144). Also, 69 DAS events were found to be common among studies 7, 4 and 3. In Fig. 2, UpSet plot represents the intersection between the DAS events from different studies based on the rMATS results.

After filtering out the DAS events that were common in less than three studies or showed ambiguous trend, 55 DAS (16 positive and 39 negative ΔPSI, as negative value indicates the exon is more skipped in fat-tailed group) corresponding to 49 genes were found. Of these, 50 DAS were assigned to SE and five to MXE. Moreover, out of 55 events, 30, 21 and four DAS were common in four, three and five studies. Totally, 14 and six significant biological processes and KEGG pathways were identified. Functional enrichment analysis revealed that the most of the enriched biological processes/pathways were related to mRNA splicing. Furthermore, some lipid metabolism-associated pathways were enriched including “Regulation of lipolysis in adipocytes” and “Lipid Catabolic Process”, which indicates the importance of AS in fat deposition in tail of sheep. Two important genes involved in lipolysis were LIPE and PLIN1 that showed negative ΔPSI (exon is most skipped in fat-tailed vs thin-tailed breeds). A list of the common DAS events across different studies identified by rMATS and their functional enrichment analysis results are provided in Supplementary File S3.

### Identification of DAS events by Whippet

**Figure 3:**
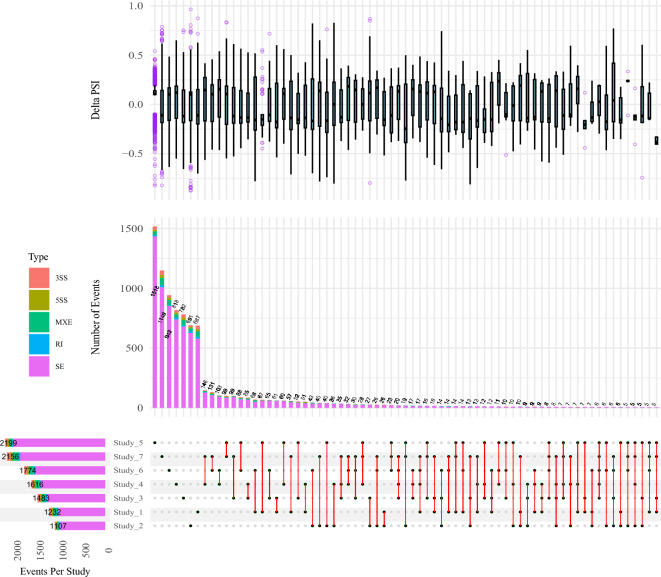
UpSet plot of the common DAS events among the different studies identified by Whippet. The box plot indicates the distribution of ΔPSI in each group. The bar plot in the middle section indicates the number of event types shared by specific studies. The horizontal bar chart at the bottom left represents the total number of events found in each study. An observation threshold of five is used to exclude low-count subsets.

On the other hand, 11,569 DAS were identified by Whippet tool including 10,118 SE, 607 MXE, 404 3SS, 248 5SS and 192 RI events. Study 5 included the largest number of DAS events (2199), followed by study 7, study 6, study 4, study 3, study 1 and study 2. SE and RI events were the most and least abundant DAS in both tools, respectively. The highest number of common DAS events was identified by Whippet between study 4 and 7 (146); and study 6 and 7 (131). Similar to the results of rMATS, the highest number of DAS events among three studies were found among studies 7, 4 and 3 (35) (Fig. 3).

In total, 215 DAS events (92 positive and 123 negative ΔPSI, as negative value indicates the exon is more skipped in fat-tailed group) met the stringent filtering criteria (be common in at last three studies with the same ΔPSI trend), and were related to 189 genes. Most of DAS events were SE (176), followed by MXE (19), 3SS (11), RI (5) and 5SS (4). Out of 215 events, 5, 28, 75 and 107 DAS were common in six, five, four and three studies. The results of functional enrichment analysis showed 19 significant biological processes terms, which were related to “Sodium Ion Transport” as well as “mRNA splicing”. Complete results of the common DAS events across different studies identified by Whippet and their functional enrichment analysis can be found in Supplementary File S4.

### Common DAS events between both tools

**Figure 4:**
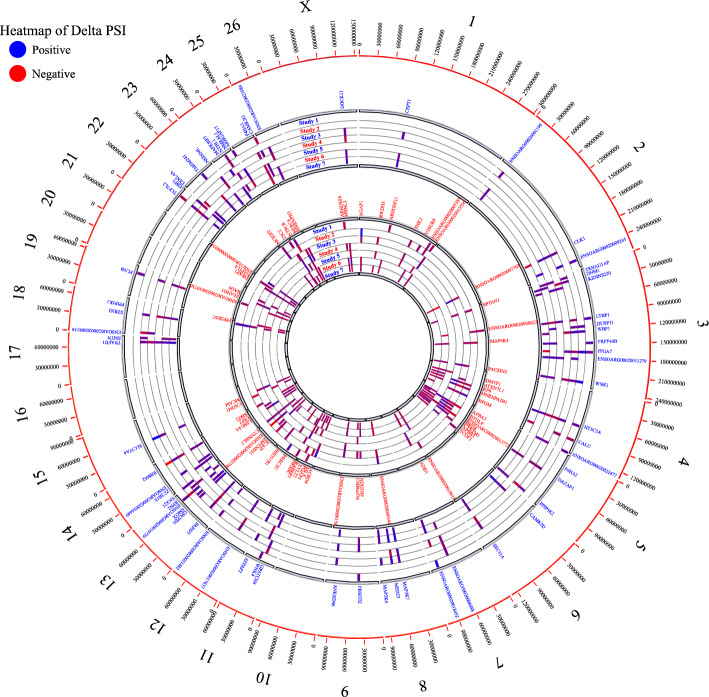
Circos plot of the genomic distributions of the final DAS, common DAS events between both tools. The outermost ring shows chromosome numbers. The position of the DAS with positive (blue) and negative ΔPSI (red) are shown in the first and third inner white rings, respectively. The heatmap in the second and fourth inner rings display the ΔPSI of each DAS with positive (blue) and negative ΔPSI (red) in different studies, respectively (studies from the outside: Study 1, Study 2, Study 3, Study 4, Study 5, Study 6 and Study 7). Positive ΔPSI indicates greater inclusion and negative ΔPSI equal decreased inclusion in fat-tailed breeds.

The DAS events that showed the same ΔPSI trend (up or down) in most of the datasets (at least three datasets) and detected by both tools, were finally considered as stable DAS events. Following these filtering criteria, 130 DAS events corresponding to 124 genes were detected, which all were related to SE class. Study 7 was characterized by the highest number of DAS events (110), followed by Study 6 (98), Study 3 (93), Study 4 (61), Study 5 (47), Study 2 (41) and Study 1 (32). Altogether, 61 statistically significant SE events with positive ΔPSI in fat-tailed breed and 69 SE events with decreased inclusion levels in thin-tailed breeds were identified by both tools and in at least in three datasets. For example, ΔPSI of SIRT2 gene for SE event is negative and indicates that the exon is more skipped in fat-tailed vs thin-tailed breeds (Supplementary File S5). A Circos plot of the genomic landscape of the common DAS along with heatmap related to their ΔPSI is presented in Fig. 4.

**Figure 5:**
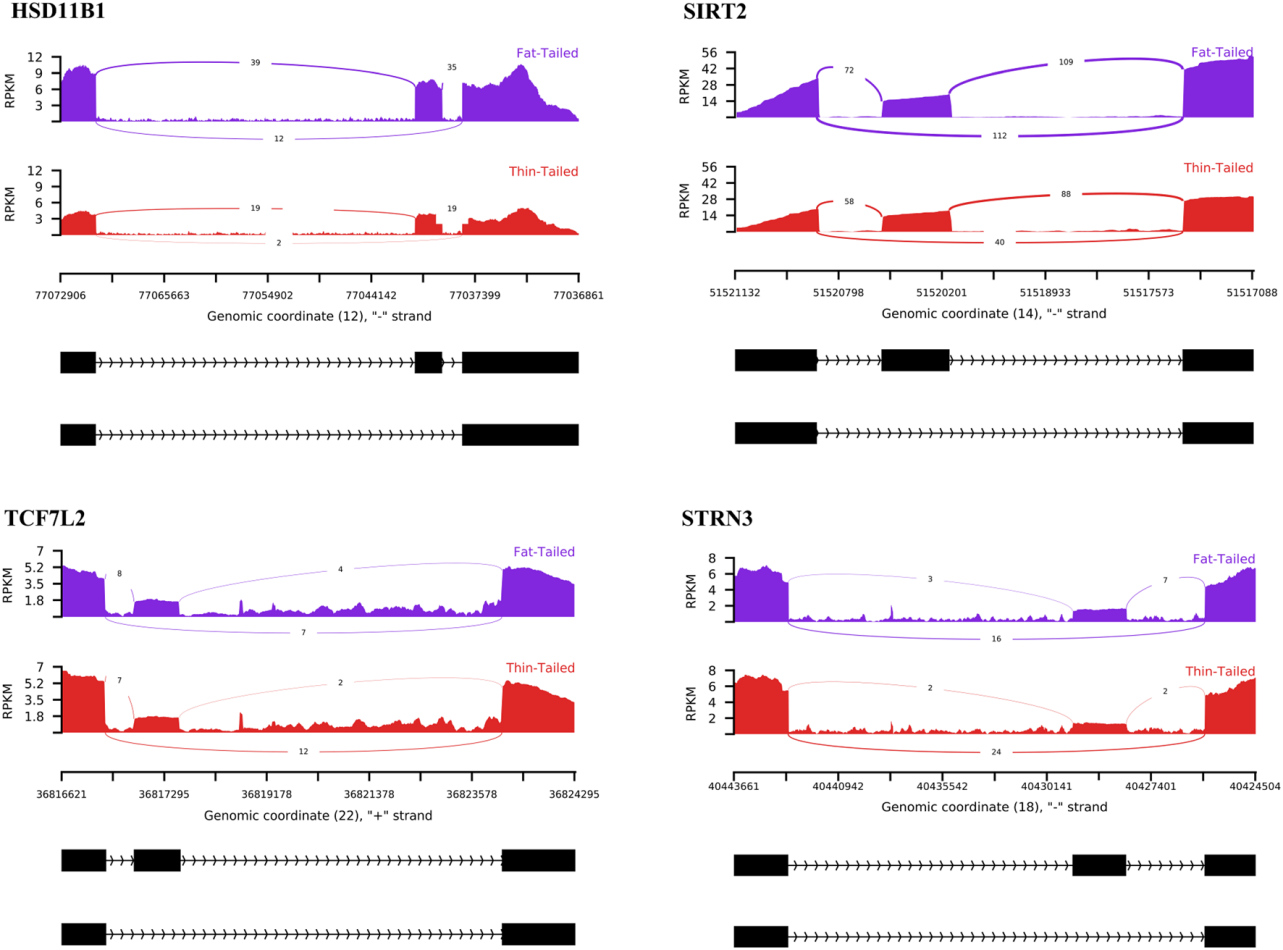
Sashimi plot of SE event within the HSD11B1, SIRT2, TCF7L2 and STRN3 genes. The blue and red tracks represent fat- and thin-tailed breeds, respectively. To provide the flexibility to compare the two groups of samples, biological replications of each group are averaged according to –group-info parameter of rmats2sashimiplot tool. Numbers on curved lines indicate average counts of the biological replications of all the samples related to different studies. The average number of junction-spanning reads of each group is clearly higher in fat-tailed than thin-tailed breeds in HSD11B1 and SIRT2 genes and vice versa for the other two genes. The bottom black tracks show the genomic localization of splicing events.

**Figure 6:**
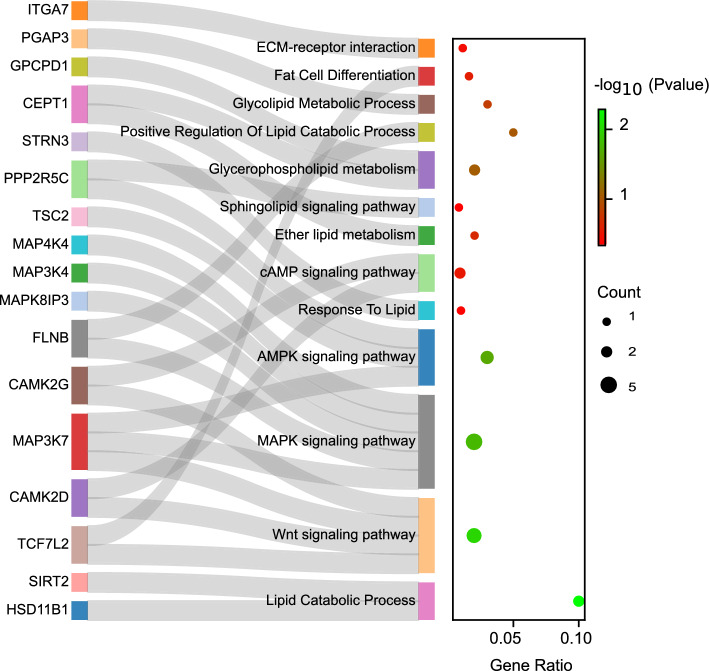
Sankey plot of the association between the DAS genes and the functional terms related to fat metabolism. The gene ratio indicates the number of DAS genes associated with the GO term or KEGG pathway divided by the total number of genes of genome that are annotated to the relevant functional terms. Count represents the number of DAS genes associated with the GO term or KEGG pathway.

To have a comprehensive understanding of the biological functions of these genes, functional enrichment analysis was performed and 22 biological processes and two KEGG pathways were significantly enriched (Supplementary File S5). Of these, “mRNA Splicing” and “Spliceosome” were the most enriched biological process and pathways, respectively. Moreover, enrichment analysis indicated the involvement of the genes in “Lipid Catabolic Process”. Two important genes related to this term were HSD11B1 and SIRT2 (Fig. 5). The exons of the both genes were more skipped in the fat-tailed breeds, as SIRT2 showed a lower exon inclusion in the fat-tailed breeds (average ΔPSI in three studies =  − 0.25) in comparison to HSD11B1 (average ΔPSI in three studies =  − 0.12). Three important significant pathways, at the p-value level (< 0.05), were found to be related to lipid metabolism including “AMPK signaling pathway”, “MAPK signaling pathway” and “Wnt signaling pathway”. Furthermore, several genes were found that were annotated to fat metabolism associated functions such as CEPT1, PPP2R5C, GPCPD1, FLNB, PGAP3, STRN3, TCF7L2 and ITGA7. The Sankey plot in Fig. 6 shows the associations of these genes with the relevant functional terms.

On the other hand, a significant PPI network (p-value < 0.001), of the DAS genes, contains 105 nodes and 54 edges was constructed using STRING database. Further annotating the DAS genes with AnimalTFDB database (version 3.0)^29^ revealed that five of 124 genes were TFs including USF2, EBF3, MLX, TCF7L2 and DMTF1. GO analysis of these genes results in 43 significant terms including “Fat Cell Differentiation” (Supplementary File S5).

## Discussion

Recent studies have shown that AS is more frequent and complex than initially thought, and AS is likely to be involved in shaping the variations among species and breeds. Previous studies have mainly focused on the transcriptional level to explore the genetic mechanisms behind the fat-tail formation in sheep^6,7,9–11,16^, leaving the other important post-transcriptional regulation mechanisms, such as AS, that may also contribute to the evolution of biological complexity and phenotypic diversity in sheep fat-tail. Here, taking advantage of RNA-Seq method, a large number of samples related to seven studies, with the same purpose, were applied to elucidate the potential roles of AS regulation in forming the fat-tail of sheep.

In total, number of the identified DAS by Whippet (11,569 events) was more than two times than rMATS (4575 events), which is in line with the previous study that reported the same ratio^30^. This finding can be attributed to the different algorithm behind the software. Whippet uses splice graph approach to represent each transcript and dose not generate p-values, which can capture more DAS events^31^. On the other hand, rMATS uses a hierarchical framework to generates inclusion levels based on the number of read counts spanning the splice junctions and provides adjusted p-values for each DAS event^32^. The annotated sheep genome has 20,137 protein coding genes (ENSEMBL release 106), 18,221 was multi-exon genes, which are exposed to AS. Out of these, 2344 and 4545 genes were found to be DAS using rMATS and Whippet, respectively. This large number of DAS events suggests that AS can be considered as a major mechanism for regulating gene expression and protein diversity in fat-tail of sheep. Nonetheless considering each of these tools separately can be led to false positive results. Hence, multiple filters with stringent double verification (intersection of rMATS and Whippet) were implemented in the bioinformatics pipeline and permitted the identification of 130 DAS events (SE) between fat- and thin-tailed sheep breeds. This concurs well with the global AS pattern of mammals, as SE event is the most common type of AS (~ 40%)^33^. For instance, SE was the most enriched event in muscle^25^ and adipose^26^ tissue of small-tailed Han than Dorset sheep breeds. However, the other AS events were not found, which is attributable to the applied stringent filtering approach. In other words, it is preferred to reduces the rate of false positive results to find the most reliable DAS genes, as may give rise to false negative findings. In a previous study, rMATS was used to investigate the splicing profiles of genes in adipose tissue of two breeds of sheep: polled Dorset and small tail Han^26^. Totally, 411 DAS events related to 364 genes were identified, of which eight genes were common with the current study results. This low consistency, can be largely attributed to a high rate of the false positive results in Miao et al. study, as they applied one AS event detection tool on one RNA-Seq dataset. Furthermore, they used adipose tissue, as fat-tail tissue was used in the present study.

If AS plays an important role in regulating the fat deposition in the tail of sheep, it can be expected to find some DAS gens which are involved in lipid metabolism related processes. First, to compare the identified DAS genes with the reported DEGs in the previous cohort studies, a comprehensive literature review was performed. It is of interest to note that 50 DAS genes were reported as DEGs in these studies^6,7,9–11,16^. These genes are listed in Supplementary File S5. Moreover, Functional analysis of the final DAS genes revealed DAS genes involvement in the relevant biological/pathways to fat metabolism including “Lipid Catabolic Process”, “Fat Cell Differentiation”, “AMPK signaling pathway”, “MAPK signaling pathway” and “Wnt signaling pathway”. Involvement of Wnt pathway^34^ and MAPK pathway^35^ in adipogenesis; and AMPK pathway^36^ in lipid metabolism have been demonstrated. Of DAS genes, HSD11B1, SIRT2, STRN3 and TCF7L2 were annotated to be directly involved in lipid metabolism (Fig. 5). In contrast to HSD11B1 and SIRT2 genes, the exons of STRN3 and TCF7L2 were more skipped in the thin-tailed breeds. HSD11B1 as a member of family of short-chain dehydrogenases/reductases, encodes hydroxysteroid (11-beta) dehydrogenase type 1 that catalyze the conversion of inactive hormone cortisone into its active form cortisol^37^. Role of HSD11B1 in the accumulation of abdominal fat has been demonstrated^38^. It is reported that HSD11B1 can affects glucose uptake by adipocytes and leads to obesity in pig^39^. Overexpression of this gene in adipocytes of mouse models is reported to be associated with the development of central obesity^40^. In this regard, HSD11B1 knockout mice were protected against the development of obesity after high-fat diet exposure^41^. HSD11B1 was identified as differentially expressed gene according to my previous study on the same datasets^6^, which was up-regulated in fat-tailed breeds. Also, this gene was reported as DEG in the other previous studies that investigated the expression pattern of genes in fat-tail tissue of fat- and thin-tailed sheep breeds^8,16^. Interestingly, DAS has been previously confirmed in adipose tissue of sheep for HSD11B1^26^, which reinforce its importance in fat-tail tissue biology. More details of HSD11B1 gene and its important roles in development of obesity and body fat distribution has been comprehensively reviewed by Nascimento et al.^37^. SIRT2 is a member of the family of sirtuins, a family of NAD + -dependent deacetylases, that regulate some important biological processes like lipid metabolism^42^. Potential function of this gene in fat deposition in pig is reported^43^. It has been shown that SIRT2 is involved in the differentiation of rodent adipocyte precursors^44^. Moreover, down regulation of SIRT2 in human stem cells promoted the expansion of visceral adipose tissue^45^. In a recent study, SIRT2 was reported as a candidate that affect the lipid metabolism in cattle^22^. Recently, as a new idea for the regulation of lipid metabolism, sirtuins are discussed as regulators in lipid autophagy^42^. All these findings lead us to conclude that sirtuins family members, specially SIRT2, can be considered as novel candidates in modulating the gene expression in fa-tail of sheep. It is worthy of notice that SIRT2 was identified as DEG in the previous studies that compared the fat-tail tissue of fat- and thin-tailed sheep breeds^10,16^.

The other important DAS gene in the present study was TCF7L2 that encodes a TF that plays a key role in the Wnt signaling pathway, which is involved in many biological processes such as lipid metabolism, regulating body mass and glucose metabolism^46^. This gene is known to be an important regulator of triglyceride accumulation through Wnt signaling pathway^36^. It is well known that TCF7L2 is involved in adipocyte differentiation and adipogenesis and its knockout promotes adipogenesis^47^ and impairs adipocyte differentiation^48^. Moreover, it is demonstrated that Wnt signal pathway plays important roles in mouse obesity^49^. The STRN3 gene encodes a protein called striatin-3, which is a member of the striatin family of proteins that are involved in various cellular functions, such as signal transduction, cytoskeleton organization, cell motility^50^ and lipid metabolism^51^. Since, these genes were identified as DAS in the present study and based on the evidence from the previous studies described above, it is possible to candidate them as potential regulators of sheep fat-tail development.

Looking specifically at the other functional terms of the DAS genes, “AMPK signaling pathway”, “MAPK signaling pathway”, “cAMP signaling pathway” and “Wnt signaling pathway”, revealed important genes involved in fat metabolism (Fig. 6). Two members of calcium/calmodulin-dependent protein kinases II (CAMK2) family including CAMK2D and KAMK2G were identified as DAS genes. A recent study has shown that CAMK2 regulates adipocyte lipolysis and its activity is increased in obese mice adipose tissue, which makes it a critical component of metabolic regulation in obesity^52^. Vital role of MAP3K4 (as an important member of the MAPK signaling pathway) in the hepatic lipogenesis of non-alcoholic fatty liver disease has been well described^53^. The expression of other important member of MAPK in the final DAS genes, MAP4K4, is reported to be increased during obesity, which makes it an important negative regulator of adipose lipogenesis in metabolic disease^54^. FLNB, as a member of the filamin family, is suggested to be strongly related to the differentiation of fat cells in Chickens^55^. These findings along with the previous studies reinforced that these genes may be involved in morphological diversity of fat-tail among the sheep breeds. The results of the current study tempted us to speculate that AS (specially SE events) may be induced during the evolution of fat- and thin-tailed sheep breeds to increase the complexity of the transcriptome as well as the diversity of the proteome. Hence, understanding the patterns and functions of AS is crucial for revealing the molecular basis of sheep fat-tail development.

## Conclusion

While understanding the molecular pathways of fat deposition in tail of sheep is crucial, little attention has been given to AS. The present study is the first comprehensive attempt to compare the patterns of AS in fat-tail of sheep breeds with different tail shape. I was particularly interested in genes that displayed significant changes at the AS event level, because such changes are often overlooked in gene expression analysis. Applying several miscellaneous methods allowed us to obtain stringent and robust results for the identification of splicing events. Of the final set of DAS events, 69 and 61 events showed negative and positive ΔPSI, respectively, which several genes were related to fat metabolism and were suggested as possible candidates involved in the sheep fat-tail development including HSD11B1, SIRT2, STRN3 and TCF7L2. Taken together, the results of the present study indicate that it is worth shifting our focus to post-transcriptional regulations mediated by various molecular mechanisms, such as AS, to reveal mechanisms underlying fat deposition. This study provides the first evidence that AS is an important mechanism contributing to regulation of gene expression of fat deposition in tail of sheep breeds.

## Materials and methods

### RNA-seq datasets

Based on a comprehensive literature review, seven independent RNA-seq datasets of the matched fat- and thin-tailed sheep breeds were obtained from the GEO database, which met the requirements for the analysis. All the samples were collected from fat-tail tissue of the male gender and were sequenced as paired-end (Table 1). A same bioinformatic pipeline was applied to analyze each RNA-Seq dataset, explained as follow.

**Table 1:** List of the used RNA-seq datasets that compared fat- and thin-tailed sheep breeds.

Fat (Nu)*	Thin (Nu)*	Length of reads	Accession number
Lori (4)	Zel (4)	150	PRJNA508203^6^
Han-Fat (3)	Han-Thin (3)	75	PRJNA699984^8^
Lanzhou (3)	Han (3)	150	PRJNA432669^10^
DHH (3)	DDH (3)	150	PRJNA745517^9^
Hulun-Fat (3)	Hulun-Thin (3)	101	PRJNA517348^16^
Ghezel (3)	Zel (4)	150	PRJNA598581^11^
Hu (3)	Tibetan (3)	150	PRJNA792697^7^

*Name of the fat- and thin-tailed breeds (Number of biological replications). Of the nine sheep breeds, Lori, Zel and Ghezel breeds were from Iran and the other sheep breeds were from China.

### Quality control, trimming and aligning of reads

Quality of the reads, before and after trimming, were assessed using FastQC (v0.11.5) tool^56^. Trimming of poor-quality reads/bases and adapter sequences was performed using adaptive trimming algorithm of Trimmomatic (v0.38) software (TRAILING: 20 and MAXINFO: 80: 0.8)^57^. Then, clean reads were aligned to the sheep genome (Oar Rambouillet v1.0) based on STAR software (v2.7.9a)^58^. The STAR index was generated based on the Ensembl ovine GTF file (rambouillet_v1.0.106) with a sjdbOverhang of 100. Two-pass approach was applied to align the reads as follow; (1) In the first round, all the samples of each dataset were aligned to the genome and the splice junction outputs were retrieved, (2) All the junction files of the previous round were used as annotated junctions for the second round to improve the accuracy and sensitivity of read alignment and detect more spliced reads mapped to novel junctions.

### Identification of AS events

Generally, five types of AS are commonly studied, including SE, IR, 5SS, 3SS and MXE. There are several software developed to identify DAS event based on these five events, as AS events abundance are compared across two or more groups. Benchmark studies suggested that to decrease the tool-specific false positives as well as to increases the validity of downstream analyses, it is better to overlap the results of two or more software^59^. Here, to minimize the potential false positive results, two well-known tools with different background rMATS^32^ and Whippet^31^ were applied and overlapping results of both tools were taken into consideration. To this end, identification and quantification of AS events (SE, IR, 5SS, 3SS and MXE) as well as detection of DAS events between the two groups (fat- and thin-tailed breeds) were performed by both tools for each dataset, separately. Then, all the results of the seven datasets were integrated and DAS events that were observed in at least three datasets with the same ΔPSI trend (percent spliced in), up or down, were considered as reliable DAS, which may be potential candidate involved in fat-tail formation in sheep. It is worth emphasizing that a stable DAS have to be detected by both tools. For example, if a DAS was detected in three studies by rMATS with the same ΔPSI trend, it has to be identified in at least one study by Whippet. This level of stringency was employed to increase the robustness and reliability of the results.

### rMATS (replicate multivariate analysis of transcript splicing)

Using a hierarchical framework, rMATS simultaneously model the variability among replicates and compute the inclusion level from two-groups RNA-seq data to identify the five classical AS events. rMATS quantify AS events as PSI, which is a ratio (the number of reads specific to exon inclusion isoform divided by the sum of reads specific to exon inclusion and exclusion isoforms) that indicates how efficiently reads of interest are spliced into transcripts. In this regard, ΔPSI is the difference between the PSI values of the two groups under study. Furthermore, P-value and false discovery rate (FDR) is calculated based on likelihood-ratio test to assess the statistical significance of the ΔPSI between two groups^32^. Here, the read alignments obtained from the above-mentioned two-pass approach was provided as input to rMATS (v4.1.2). Both exon–exon junction reads and the reads mapped to the exon body were used in the analyses (JCEC). In each dataset, AS events exhibiting a significant difference between fat- and thin-tailed samples with an FDR < 0.05 were considered as DAS.

### Whippet

Based on gene annotation and genome files, Whippet builds contiguous splice graphs (CSGs) to represent each transcript. Then, to quantify splicing events, reads directly align to the CSGs. Whippet considers splicing events as nodes, as each node corresponds to an exonic region of the gene, and the incoming and outgoing edges to each node define the set of reads supporting its inclusion and exclusion, respectively^31^. Like rMATS, PSI is defined as a measure of the relative abundance of transcripts which contains the target exon over the relative abundance of all transcripts. This ratio can be calculated through number of the paths supporting the inclusion of the node divided by the total number of the paths supporting both the inclusion and exclusion of that node^31^. Here, Whippet v1.6.2 was applied to quantify read data and identify DAS between two breeds in each dataset. To create the splice graphs, sheep genome (Oar Rambouillet v1.0) and Ensembl ovine GTF file (rambouillet_v1.0.106) were used. Whippet was run with the “biascorrect” option to consider GC-content and 5’ sequence bias correction. To generate more accurate results, events with associated posterior probability > 0.8 and |ΔPSI| ≥ 0.1 were considered as DAS.

### Functional analysis

To identify the main biological processes or pathways that the genes with stable DAS may be involved in, functional enrichment analysis was performed. All these genes were used for gene ontology (biological process) and KEGG (Kyoto Encyclopedia of Genes and Genomes) analysis using EnrichR tool^60^. The terms with p-value < 0.01 and FDR < 0.2 were considered statistically significant. Moreover, these genes were subjected to protein–protein interaction (PPI) network analysis using STRING database to determine whether they are members of functional and significant interaction networks. Also, transcription factors (TFs) that were presented in the final DAS genes were identified according to AnimalTFDB database (version 3.0)^29^.

### Visualization of the results

Splicing events were visualized using rmats2sashimiplot (https://github.com/Xinglab/rmats2sashimiplot) R package based on the inclusion levels obtained from rMATS. ComplexUpset (https://krassowski.github.io/complex-upset, v1.3.3) and interacCircos (https://github.com/mrcuizhe/interacCircos, v1.2.0) R packages were applied to construct the upset and circos plots, respectively.

### Supplementary Information

Supplementary Information 1.

Supplementary Information 2.

Supplementary Information 3.

Supplementary Information 4.

Supplementary Information 5.

## Data Availability

The datasets analyzed during the current study are available in the GEO repository of NCBI (PRJNA508203, PRJNA699984, PRJNA432669, PRJNA745517, PRJNA517348, PRJNA598581, PRJNA792697).
